# Reliability, validity and acceptability of the traditional Chinese version of the carer support needs assessment tool in Hong Kong palliative care settings

**DOI:** 10.1186/s12904-021-00852-w

**Published:** 2021-10-09

**Authors:** Hui-Lin Cheng, Doris Yin Ping Leung, Po Shan Ko, Ming Wai Chung, Wai Man Lam, Po Tin Lam, Andrew Leung Luk, Simon Ching Lam

**Affiliations:** 1 School of Nursing, The Hong Kong Polytechnic University, Hom Hung, Kowloon, Hong Kong SAR, China; 2 Nursing Services Division, United Christian Hospital, Hong Kong SAR, China; 3 Department of Medicine, Haven of Hope Hospital, Hong Kong SAR, China; 4 Department of Medicine and Geriatrics, United Christian Hospital, Hong Kong SAR, China; 5 Nethersole Institute of Continuing Holistic Health Education, Hong Kong SAR, China; 6 School of Nursing, Tung Wah College, Hong Kong SAR, China

**Keywords:** Cancer, Caregiver, Palliative care, Reliability, Validity, Acceptability, CSNAT

## Abstract

**Background:**

Among the few existing needs assessment tools for family carers, the 14-item Carer Support Needs Assessment Tool (CSNAT) is the only brief and holistic needs screening tool designed for everyday use in palliative care practices. The aim of this study was to evaluate the reliability, validity, and acceptability of the traditional Chinese version of the CSNAT in palliative care settings in Hong Kong.

**Methods:**

This adopted a cross-sectional and correlation design with repeated measures. The participants were 125 family carers of palliative cancer patients and 10 healthcare providers (HCPs) that were recruited from two local hospitals. The evaluation of psychometric properties included the following: (1) content validity through HCPs including frontline physicians, nurses, social workers, and clinical psychologists; (2) construct validity between the CSNAT items and those of the validated tools that measured caregiver burden, social support, and caregiving self-efficacy; and (3) one-week test-retest reliability in a sub-sample of 81 caregivers. The acceptability of the tool was assessed by the carers using several closed-ended questions.

**Results:**

The content validity index of the CSNAT at the scale level was 0.98. Each item of the CSNAT was significantly and moderately correlated with caregiver burden (Spearman’s *r* = 0.24 to 0.50) and caregiving self-efficacy (*r* = − 0.21 to − 0.52), but not for social support. All CSNAT items had fair to moderate test-retest reliability (weighted kappa = 0.21 to 0.48), with the exception of two items “managing your relatives’ symptoms, including giving medicines” and “having time for yourself in the day”.

Regarding the acceptability of the CSNAT, almost all HCPs were willing to use the CSNAT for carer assessment and support. 89.6% of the carers demonstrated a comprehensibility of the CSNAT tool and 92.9% felt comfortable answering the questions. Around 90% of the carers agreed to use the tool for screening, discussing needs, and making referrals.

**Conclusion:**

The traditional Chinese version of the CSNAT is a tool with high validity and acceptability and adequate reliability that measures family carers’ support needs, which should be considered for wide application in local palliative care practices.

## Background

The World Health Organization has recognized palliative care as an approach that aims to maximize the quality of life of patients with advanced and incurable diseases and their families [1]. The core essence of palliative care is the provision of holistic, person-centered care that addresses various dimensions of support needs among individuals [2]. While palliative care services have typically focused on patients, relatively little attention has been directed to family caregivers, who are profoundly challenged by their caregiving roles [3]. As caring for terminally ill patients at home is physically and emotionally demanding, most family carers experience higher levels of stress, and thus have unmet support needs that should be targeted for intervention [4–6]. To provide appropriate palliative care for this population, there is an urgent need for validated assessment tools to identify their support needs.

The term “needs” is generally viewed as actions or resources that individuals require from professional help to attain optimal well-being [7]. A recent systematic review identified seven unmet support needs domains that are common for informal carers of palliative care patients: information, physical, psychological, financial, care services, spiritual, and social needs; however, these domains are seldom fully covered by a single tool [8]. Several comprehensive instruments measuring the carers’ needs within the context of palliative care are found, for example, the Problems and Needs in Palliative Care and the Supportive Care Needs Survey—Partners and Caregivers, which are typically lengthy, with the number of items ranging from 40 to 67 [9, 10]. Nonetheless, there is a significant lack of holistic assessment tools that have been developed to aid healthcare providers (HCPs) in quickly identifying their support needs and prioritizing core needs for effective support or timely referrals [11].

Among the few existing needs screening tools for carers, the 14-item Carer Support Needs Assessment Tool (CSNAT) is the only very brief but holistic needs screening tool for practical use in palliative care [12–14]. Building on the literature and qualitative work, the CSNAT was developed to measure carers’ support needs in multiple domains, including physical, practical, social, financial, psychological, and spiritual [8, 12]. It has been validated in several samples of family carers caring for relatives primarily diagnosed with advanced cancer, and it demonstrated good face, content, and construct validity as well as test-retest reliability [13, 14]. Since the CSNAT tool was constructed as a screening tool rather than a scale, with each item indicating a dimension of holistic needs, only the item score is calculated, not the total score [12–14]. The CSNAT was developed as part of an intervention facilitated by HCPs to identify and address carers’ support needs, which has been increasingly integrated into palliative care services in some European countries [15, 16].

Though the CSNAT was developed in 2013, it has been translated and validated into different languages, including German, Swedish and Simplified Chinese [14, 17, 18]. The CSNAT has been translated into traditional Chinese following forward and backward translation and applied in family carers of older adults [19]; however it has not yet been validated for family carers of palliative cancer patients. Prior to its large application in clinical practice, a methodologically rigorous validation of the tool is therefore warranted. More importantly, the acceptability of the CSNAT tool by caregivers has been less examined and is exclusively based on qualitative data [13, 17]. Acceptability is defined as the extent to which carers and HCPs can complete and/or use the questionnaire in clinical practice [20]. In view of carers’ complex support needs, a tool with high carer acceptability would increase its utility in the local context. Prior to validate the CSNAT tool in local context, the tool was reviewed and judged as having a high face validity by four team members who are experts in the field of palliative care and family caregiving. Therefore, this study aimed to test the reliability, validity, and acceptability of the traditional Chinese version of the CSNAT for carers of palliative cancer patients in Hong Kong.

## Methods

### Study design and setting

The present study adopted a cross-sectional and correlation design with repeated measures. The study setting were palliative care units of the two palliative care hospitals in Hong Kong.

### Participants

The participants were recruited by convenience sampling from December 2019 to December 2020. The participants included HCPs (to test content validity and acceptability) and carers of palliative cancer patients (to test construct validity, test-retest reliability, and acceptability). A sub-sample of caregivers volunteered to complete the CSNAT again after 1 week [14]. In this study, a family carer is defined as a family member who provides regular care or assistance for a person receiving palliative care [7].

Caregivers were included if they were: (1) adults aged 18+ years old; (2) family members taking care of palliative cancer patients at home; and (3) able to communicate in Cantonese. Caregiver exclusion criteria included: (1) paid caregivers; and (2) mentally incapable of completing the questionnaire (Hong Kong version of the Montreal Cognitive Assessment score < 22) [21]. Eligibility criteria for the HCPs included any physician, nurse, and other allied healthcare staff with direct experience in supporting carers of palliative cancer patients.

The sample sizes for the HCPs and carers were estimated based on different psychometric property testing. For content validity testing, according to the international guidelines, at least 10 experts are sufficient to achieve consensus on establishing content validity [22]. Ten HCP experts were selected based on their positions and varying years of working experience with family carers of palliative cancer patients, who were frontline physicians, nurses, social workers, and clinical psychologists. To test the psychometric properties of the tool, at least 123 caregivers would be recruited to reach 80% power and to show construct validity, assuming that the observed Spearman’s Rho (*r*) correlation coefficient was 0.25 based on two published validation reports of the CSNAT, at a significance level of 5% for the two-tailed tests [13, 14]. To evaluate the one-week test-retest reliability of the CSNAT, assuming that the weighted quadratic kappa (Kw) value was 0.40 (moderate) for the categorical data from the two-tailed tests [14], at least 50 carers were required to achieve a power of 0.80 [23].

### Caregiver surveys

#### Carer support needs assessment tool

The carers’ support needs were measured using the traditional Chinese version of the CSNAT [19]. The tool has 14 items and encompasses two broad categories: “support to help carers provide care” and “more direct support for the carers themselves.” The tool also includes one optional additional question to capture any “other support need” that was not covered. Each item is rated using a 4-point scale (0 = “no”, 1=“a little more”, 2=“quite a bit more”, 3=“very much more”), with a score of ≥1 indicating the presence of more needs.

#### Caregiver strain index

Caregiver burden was assessed using the 13-item traditional Chinese version of the Caregiver Strain Index (C-M-CSI) [24], which has been widely used in family caregiving research on palliative care [25, 26]. Each item is rated as either a yes (“1”) or no (“0”) response. The total score is between 0 and 13, with a higher score indicating greater caregiver burden. The C-M-CSI has reported excellent reliability (Cronbach’s alpha coefficient = 0.91) and has established concurrent validity with the Caregiver Burden Inventory (*r* = 0.78) [24]. In this study, the scale’s Cronbach’s alpha coefficient was 0.84.

#### Social support questionnaire

The six-item traditional Chinese version of the Social Support Questionnaire (SSQ-6) was used to measure perceived social support [27, 28]. Each item consists of two parts, including the number of supportive persons and satisfaction with the social support received. A high score indicates a higher level of social support. The psychometric properties of the traditional Chinese version of the SSQ-6 have been reported, with Cronbach’s alphas exceeding 0.90 [28]. In this study, the Cronbach’s alpha coefficients of the scale’s subscales were 0.89 and 0.97, respectively.

#### Caregiver Inventory-18

The 18-item traditional Chinese version of the Caregiver Inventory (C-CGI-18) was used to assess caregiving self-efficacy in palliative care [29, 30]. It consists of three domains: “care of the care recipient,” “managing information and self-care,” and “managing emotional interactions with the care recipient.” Each item is scored using a 9-point scale, from 1 (“not at all”) to 9 (“totally confident”). The instrument has been successfully validated among local carers of patients with palliative care needs [30]. The C-CGI-18 has reported good test-retest reliability (*r* = 0.71 to 0.76), internal consistency reliability (Cronbach’s alpha = 0.84 to 0.90), convergent validity, and construct validity [30]. In this study, the scale’s Cronbach’s alpha coefficients were 0.79 to 0.83.

#### Carer acceptability questionnaire

A self-developed questionnaire was used to evaluate the carers’ acceptability of the CSNAT as a routine assessment in palliative care. Five questions are included in the questionnaire, which were aligned with the essential steps of the CSNAT intervention [15]: (1) comprehensibility of the tool; (2) comfortable answering the questions in the tool; (3) willing to be screened for support needs using this tool; (4) willing to discuss support needs with HCPs; and (5) expect that HCPs can provide direct support or refer resources for help. Each question is rated on a 6-point Likert scale, from 1 (“strongly disagree”) to 6 (“strongly agree”).

Additionally, socio-demographic data were collected from the cares, including age, gender, marital status, employment status, education level, income, relationship with the patient, duration of caregiving, and living arrangement.

### Healthcare provider survey

The HCP survey was designed to assess the content validity and acceptability of the CSNAT. For content validity testing, the content relevancy of each CSNAT item was measured using a 4-point Likert scale, from 1 (“not relevant”) to 4 (“highly relevant”). For the acceptability of the CSNAT from HCP perspectives, three items are designed based on the key steps of the CSNAT intervention [13], including (1) perceived usefulness of the tool; (2) willing to use the tool for screening; and (3) willing to discuss support needs with carers. Each question is rated on a six-point Likert Scale, ranging from 1 (strongly disagree) to 6 (strongly agree) and a higher score indicates a higher level of acceptability.

### Procedures

Carers were approached by a research assistant when they were accompanying palliative cancer patients to outpatient units or visiting palliative cancer patients in inpatient units in the two hospitals. If the carers showed an interest in participating in the study, the research assistant conducted screening for their eligibility. Once they were confirmed as eligible, the research assistant provided them with the information sheet and explained the procedures of the study. The participants’ written informed consent forms were collected before data collection. The carers were then asked to fill out the surveys (including the CSNAT) by themselves or as administered by the researcher using a pen-and-paper mode in the hospital settings. A sub-sample of 81 participants volunteered to complete the CSNAT tool again 1 week later through structured telephone interviews following astandardized interview script. The carer survey was pilot-tested on 10 carers and no comprehensibility problems were reported. Regarding the HCPs, one research team member contacted potential participants for participation. Once they agreed to join the study, they were asked to return their signed consent form along with the completed surveys within 2 weeks.

### Data analysis

Data were entered and analyzed using SPSS 22.0. Descriptive statistics were used to describe the participants’ profiles and study variables, including acceptability. The mean scores and standard deviations (SD) for the continuous variables and frequency and the percentages for the categorical variables were reported. All inferential tests were two-tailed, with a *p* value set at < 0.05.

#### Data quality

The percentage of missing data as well as the extent of ceiling and floor effects were calculated to evaluate data quality. A cut-off point of 15% was used as a criterion for determining the presence of ceiling or floor effects based on the proportions of the participants’ highest and lowest scores, respectively [31].

#### Test-retest reliability

The CSNAT tool is a screening tool, so the total scale was not calculated [13]. Test-retest reliability for each CSNAT item was calculated using weighted quadratic kappa (Kw). The kappa coefficient was interpreted with the following criteria: ≤0.20 (slight), 0.21 to 0.40 (fair), 0.41 to 0.60 (moderate), 0.61 to 0.80 (substantial), and > 0.80 (almost perfect) [32].

#### Construct validity

Each CSNAT item with external constructs, including caregiver burden, social support, and caregiving self-efficacy, was evaluated using Spearman’s correlation analyses (*r*). It was hypothesized that the CSNAT items would positively correlate with caregiver burden [13, 14], and negatively correlate with social support and caregiving self-efficacy [8, 33, 34]. A correlation coefficient ≥ 0.30 supported the construct validity of the tool [35].

#### Content validity

The content validity index (CVI) was calculated by the proportion of HCP experts who gave a rating of 3 or 4. Both the item (I-CVI) and scale (S-CVI) levels of the CVI were calculated. If at least nine experts were invited, an acceptable CVI was ≥0.78 [36].

### Ethical considerations

The team validated the traditional Chinese version of the CSNAT with permission from the original developer. Ethical approval was obtained from the researcher’s university and the two hospitals involved in the study. Written consent was collected from each participant. The personal data of the participants were kept strictly confidential and anonymized for data analysis. We ensured the participants’ right to withdraw from research at any time.

## Results

### Participants’ characteristics

Table 1 displays the characteristics of the participants. Of the 168 carers approached, 125 (74.4%) agreed to join the study. Of those who refused to participate, 43 gave the following reasons: no interest (*n* = 16), not a primary caregiver (*n* = 11), no time (*n* = 9), not in the mood (*n* = 2), response burden (*n* = 2), and others, such as language barrier (*n* = 3). Among the recruited carers who completed the baseline assessments, a subsample of 81 proceeded with the second measurement of the CSNAT within 1 week.

**Table 1:** Sample characteristics (*n* = 125 caregivers and 10 healthcare providers )

Characteristics	Mean (sd)/n(%)	Range
Carers		
Age	54.5(14.3)	23–84
Duration of caregiving (hours/week)	56.3(59.4)	2–168
Gender		%
Male	52(41.6)	
Female	73(58.4)	
Marital status		
Single	22(17.6)	
Married	95(76.0)	
Widowed/separated/divorced	8(6.4)	
Employment status		
Full-time	45(36.0)	
Part-time	11(8.8)	
Unemployment	25(20.0)	
Retired	34(27.2)	
Others (e.g. free-lance)	10(8.0)	
Education		
Elementary school and less	31(24.8)	
High school	60(48.0)	
Colleague and above	32(27.2)	
Monthly income (HKD)		
< 5000	54(43.2)	
5000 -19,999	35(28.0)	
20,000-49,999	29(23.2)	
> 50,000	7(5.6)	
Relationship with the patient		
Spouse/partner	39(31.2)	
Children	60(48.0)	
Siblings	7(5.0)	
Parents	14(11.2)	
Others (e.g. grand-daughter and niece)	5(4.0)	
Living with the patient		
Yes	77(61.6)	
No	48(38.4)	
Healthcare providers	Mean (sd)	Range
Year of working experience	14.4(8.9)	4–33
Age	n	%
21–30	1(10.0)	
31–40	5(50.0)	
41–50	2(20.0)	
51–60	2(20.0)	
Gender		
Male	2(20.0)	
Female	8(80.0)	
Occupation		
Nurse	2(20.0)	
Doctor	2(20.0)	
Social worker	4(40.0)	
Clinical psychologist	2(20.0)	
Education		
Bachelor	3(30.0)	
Master	6(60.0)	
Professional training	1(10.0)	

**Fig. 1:**
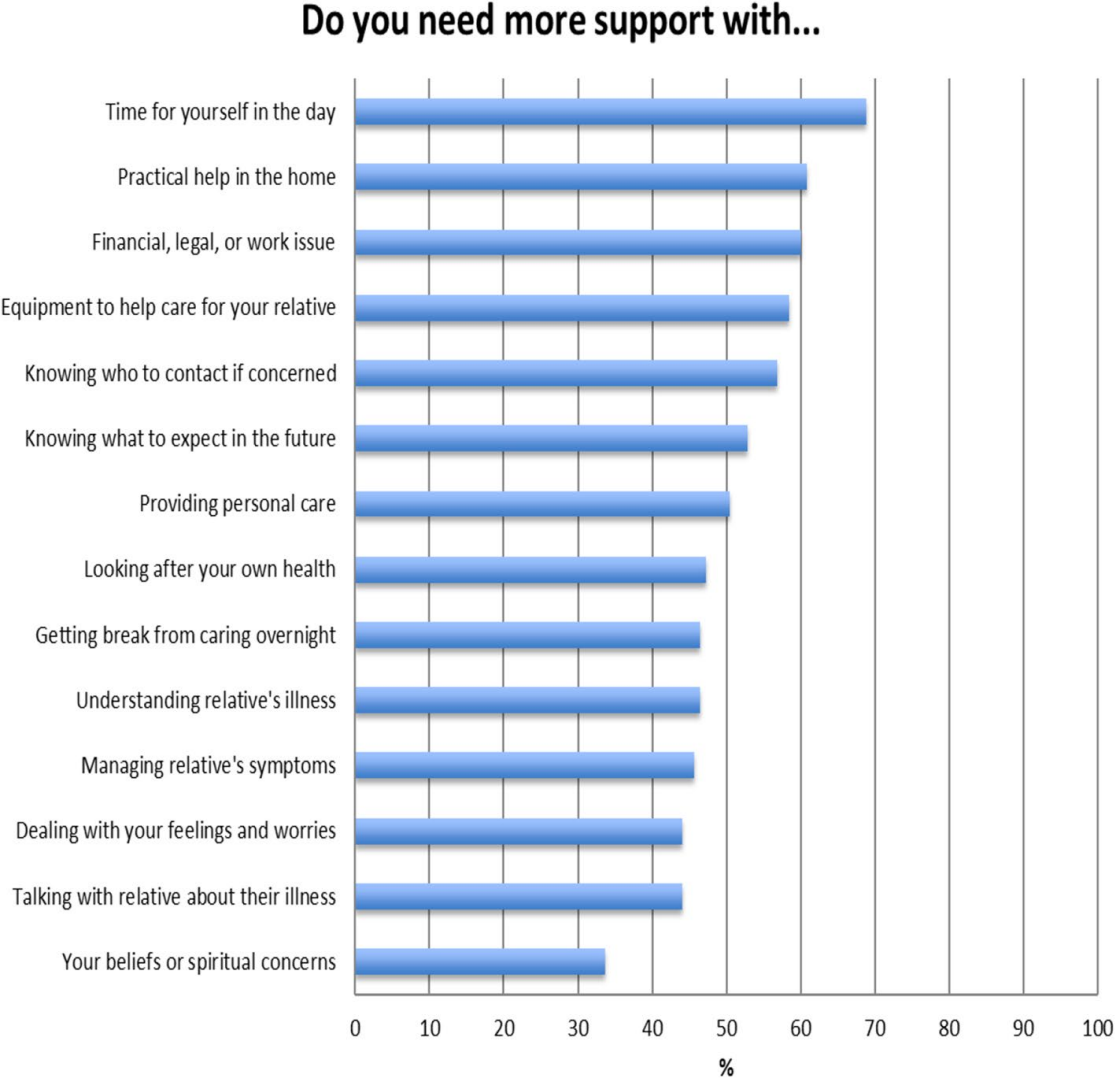
Percentage of family caregivers’ need for more support with CSNAT items (*n* = 125)

The mean age of the carers was 54.5 years old (SD = 14.3). Most of the carers were female (58.4%) and worked either full or part time (44.8%). The carers were predominantly the children (48.0%) or spouse (31.2%) of the patients. Figure 1 illustrates the percentages of the carers’ need for more support (score ≥ 1). The top three CSNAT items rated by the carers were “time for yourself in the day” (68.8%), “practical help in the home” (60.8%), and “financial, legal, or work issue” (60.0%). The least selected CSNAT item for more support was “beliefs or spiritual concerns” (33.6%). In addition to the 14 items, only two participants identified an additional need: “transportation services from home to hospitals” and “respite services in a daily care center,” respectively.

Ten HCPs were recruited for the study, which included frontline physicians, nurses, social workers, and clinical psychologists. Half of them were aged 31 to 40 years old. The majority of them were female (80%) and were master’s degree holders (60%). The mean time of the HCP’s working experience was 14.4 years, ranging from four to 33 years.

### Data quality

The data quality of the CSNAT tool is presented in Table 2. After analysis, there were no missing data from the CSNAT results. All CSNAT items were positively skewed and showed significant floor effects, with 39.2 to 66.4% of the caregivers reporting the lowest scores. The ceiling effect was identified only for the item “practical help in the home” as the highest score, representing 16% of the answers.

**Table 2:** Descriptive data of the CSNAT items among carers (*n* = 125)

Domain	Item	Score distribution, n(%)			
	Do you need more support with….	No	A little more	Quite a bit more	Very much more
Support domains to enable the carer to care	..understanding your relative’s illness?	67(53.6)	20(16.0)	25(20.0)	13(10.4)
	..managing your relative’s symptoms, including giving medicines?	68(54.4)	33(26.4)	16(12.8)	8(6.4)
	..providing personal care for your relative (eg dressing, washing, toileting)?	62(49.6)	28(22.4)	21(16.8)	14(11.2)
	..knowing who to contact if you are concerned about your relative (for a range of needs including at night)?	54(43.2)	33(26.4)	23(18.4)	15(12.0)
	..equipment to help care for your relative?	52(41.6)	36(28.8)	26(20.8)	11(8.8)
	..talking with your relative about his or her illness?	70(56.0)	33(26.4)	19(15.2)	3(2.4)
	..knowing what to expect in the future when caring for your relative?	59(47.2)	31(24.8)	26(20.8)	9(7.2)
More direct support domains for carers themseleves	..having time for yourself in the day?	39(31.2)	40(32.0)	37(29.6)	9(7.2)
	..your financial, legal or work issues?	50(40.0)	32(25.6)	32(25.6)	11(8.8)
	..dealing with your feelings and worries?	70(56.0)	30(24.0)	20(16.0)	5(4.0)
	..looking after your own health (physical problems)?	66(52.8)	36(28.8)	18(14.4)	5(4.0)
	..your beliefs or spiritual concerns?	83(66.4)	27(21.6)	10(8.0)	5(4.0)
	..practical help in the home?	49(39.2)	25(20.0)	31(24.8)	20(16.0)
	..getting a break from caring overnight?	67(53.6)	30(24.0)	14(11.2)	14(11.2)

### Test-retest reliability

As shown in Table 3, all of the CSNAT items had fair to moderate agreements (Kw = 0.21 to 0.48, *p* < 0.05 to 0.001), with the exception of two items: “managing your relative’s symptoms, including giving medicines” (Kw = 0.13) and “having time for yourself in the day” (Kw = 0.15).

**Table 3:** Test-retest reliability of the CSNAT items among caregivers (n = 125)

Domain	Items	Quadratic weighted Kappa	95% CI for Kappa	*p*-value
	Do you need more support with….			
Support domains to enable the carer to care	..understanding your relative’s illness?	0.26	0.03, 0.49	< 0.01
	..managing your relative’s symptoms, including giving medicines?	0.13	− 0.04, 0.30	0.181
	..providing personal care for your relative (eg dressing, washing, toileting)?	0.39	0.18,0.60	< 0.001
	..knowing who to contact if you are concerned about your relative (for a range of needs including at night)?	0.21	0.03, 0.40	< 0.05
	..equipment to help care for your relative?	0.41	0.20,0.61	< 0.001
	..talking with your relative about his or her illness?	0.36	0.14,058	< 0.01
	..knowing what to expect in the future when caring for your relative?	0.24	0.01,0.46	< 0.05
More direct support domains for carers themselves	..having time for yourself in the day?	0.15	−0.06,0.37	0.140
	..your financial, legal or work issues?	0.40	0.19,060	< 0.001
	..dealing with your feelings and worries?	0.48	0.27,0.70	< 0.001
	..looking after your own health (physical problems)?	0.23	0.03,0.42	< 0.05
	..your beliefs or spiritual concerns?	0.46	0.20,0.72	< 0.001
	..practical help in the home?	0.30	0.12,049	< 0.01
	..getting a break from caring overnight?	0.29	0.12,0.47	< 0.01

The following criteria used for the interpretation of the Kappa values: ≤ 0.20 (slight), 0.20–0.40 (fair), 0.41–0.60 (moderate), 0.60--0.80 (good), and > 0.80 (very good)

### Construct validity

Table 4 shows the correlations of the CSNAT items with caregiver burden, caregiving self-efficacy, and social support. The scores of each CSNAT item were significantly and moderately correlated with the C-M-CSI scores (*r* = 0.24 to 0.50, *p* < 0.01). Moderate and significant associations were found between the CSNAT items and each domain score of the C-CGI-18 scale, including “managing information” and “self-care” (*r* = − 0.22 to − 0.45, *p* < 0.05 to 0.01), “care of the care recipient” (*r* = − 0.21 to − 0.42, *p* < 0.01), and “managing emotional interaction with care recipient” (*r* = − 0.26 to − 0.52, *p* < 0.01). However, the relationships between the CSNAT item scores and the SSQ-6 scores were not significant.

**Table 4:** Spearman’s correlations between CSNAT items and standard measures of caregiver burden, social support, and caregiving self-efficacy (*n* = 125 caregivers)

Domain	Do you need more support with….	Caregiver burden	Social support		Caregiving self-efficacy		
			Support person	Support satisfaction	Managing information and self-care	Care of the care recipient	Managing emotional interaction with care recipient
Support domains to enable the carer to care	..understanding your relative’s illness?	0.24**	0.02	−0.10	−0.41**	−0.32**	−0.42**
	..managing your relative’s symptoms, including giving medicines?	0.32**	−0.02	−0.17	− 0.34**	−0.31**	− 0.48**
	..providing personal care for your relative (eg dressing, washing, toileting)?	0.39**	−0.07	−0.10	− 0.26**	−0.22**	− 0.39**
	..knowing who to contact if you are concerned about your relative (for a range of needs including at night)?	0.32**	−0.05	−0.01	− 0.36*	−0.32**	− 0.39**
	..equipment to help care for your relative?	0.39**	0.10	−0.10	−0.41**	− 0.32**	−0.54**
	..talking with your relative about his or her illness?	0.27**	−0.01	−0.16	0.44*	−0.41**	− 0.46**
	..knowing what to expect in the future when caring for your relative?	0.25**	−0.06	−0.18*	− 0.45**	−0.39**	− 0.52**
More direct support domains for carers themseleves	..having time for yourself in the day?	0.49**	−0.10	−0.03	− 0.30**	−0.32**	− 0.38**
	..your financial, legal or work issues?	0.42**	−0.06	−0.05	− 0.25**	−0.27**	− 0.30**
	..dealing with your feelings and worries?	0.43**	0.04	−0.16	−0.39**	− 0.42**	−0.41**
	..looking after your own health (physical problems)?	0.32**	0.01	−0.03	−0.29**	− 0.22**	−0.26**
	..your beliefs or spiritual concerns?	0.39**	−0.03	−0.06	− 0.38**	−0.38**	− 0.33**
	..practical help in the home?	0.49**	0.11	−0.07	−0.22*	− 0.21**	−0.39**
	..getting a break from caring overnight?	0.50**	0.11	−0.06	−0.25**	− 0.26**	−0.33**

^**^< 0.01, ^*^ < 0.05

### Content validity

The I-CVIs of the CSNAT were between 0.90 and 1.00, and the S-CVI of the CSNAT was 0.98.

### Acceptability

Table 5 illustrates the acceptability data of the CSNAT by the HCPs and carers. Nine out of the 10 HCPs were willing to use the CSNAT for carer assessment and support. Of the carers, 89.6% demonstrated a comprehensibility of the tool, and 92.9% felt comfortable answering the questions. Around 90% of the carers agreed to use the tool for screening, discussion, and referrals.

**Table 5:** Acceptability of the CSNAT among HCPs (*n* = 10) and carers (*n* = 125)

Items	N(%)
Healthcare providers	
Perceived usefulness of the tool	
Very disagree	1(10)
Agree	6(60)
Very agree	3(30)
Wiling to use the tool for screening	
Very disagree	1(10)
Slightly agree	1(10)
Agree	6(60)
Very agree	2(20)
Willing to discuss support needs with carers	
Very disagree	1(10)
Agree	6(60)
Very agree	3(30)
Carers	
Comprehensibility of the tool	
Very much disagree	1(0.8)
Disagree	2(1.6)
Slightly disagree	10(8.0)
Slightly agree	20(16.0)
Agree	52(41.6)
Very much agree	40(32.0)
Be comfortable answering the questions in the tool	
Disagree	2(1.6)
Slightly disagree	7(5.6)
Slightly agree	29(23.2)
Agree	49(39.2)
Very much agree	38(30.4)
Willing to be screened for support needs using this tool	
Disagree	2(1.6)
Slightly disagree	12(9.6)
Slightly agree	23(18.4)
Agree	52(41.6)
Very much agree	36(28.8)
Willing to discuss support needs with healthcare providers	
Disagree	2(1.6)
Slightly disagree	11(8.8)
Slightly agree	26(20.8
Agree	52(41.6)
Very much agree	34(27.2)
Expect that healthcare providers offer direct support or refer resources for help	
Very much disagree	2(1.6)
Disagree	3(2.4)
Slightly disagree	9(7.2)
Slightly agree	17(13.6)
Agree	39(31.2)
Very much agree	55(44.0)

## Discussion

The present study provided evidence of the reliability, validity, and acceptability of the traditional Chinese version of the CSNAT within the Hong Kong palliative care context. The findings indicated that the traditional Chinese version of the CSNAT had high construct validity, content validity, and acceptability as well as acceptable test-retest reliability. This tool should be considered for future use to assess the support needs of carers of palliative cancer patients using traditional Chinese characters.

In this study, no missing data from the traditional Chinese version of the CSNAT occurred. Consistent with a previous validation report in Sweden [14], the study results showed a positive skewed distribution of all the CSNAT items. It is well recognized that the presence of floor effects may lower the tool’s responsiveness to change and may fail to capture the full range of support needs [37, 38]. Limited studies have reported that the vast majority of CSNAT items did not change over time [13, 39]. Longitudinal tobit regression model can be considered to account for the presented floor effects in future studies [40].

A validation study in Sweden was the first to examine the CSNAT’s test-retest reliability, and the study reported that all the items had moderate to good agreement at baseline and 1 week later [14]. Similar results were found in this study, in that the vast majority of items met the kappa standard for determining fair to moderate test-retest reliability at the same between-assessment interval [32]. Nonetheless, two items reached poor agreement between the test and retest scores, which were “managing your relative’s symptoms, including giving medicines” and “having time for yourself in the day.” Such findings may be explained by the inconsistent data collection methods at the two assessment time points, as the second assessment used structured telephone interview, which differed from self-completed mode at baseline. Additionally, the findings may be related to the clinical instability of advanced cancer patients, which has been cited as the most significant factor that influences test-retest reliability results in palliative care studies [41]. The original validation study did not measure the test-retest reliability of the CSNAT under the same condition of assessing how carers are affected by patients’ unpredictable disease progression [13]. Additional work is needed to examine the test-retest reliability of the CSNAT in future studies.

As hypothesized, significant correlations of the CSNAT item scores with those of the C-M-CSI and C-CGI-18 were confirmed in this study, supporting the scale’s construct validity. These findings are also consistent with published studies that measured the relationships of the same constructs among carers of palliative care patients [13, 14, 18, 33]. Surprisingly, this study found that the CSNAT was not significantly associated with the SSQ-6, which was not in line with the study’s hypothesis. Previous research has shown that stronger social support was associated with less support needs among carers of advanced cancer patients [34]. The current study adopted the SSQ-6 to measure the number of supportive networks and satisfaction with support, which failed to address the functional aspect of social support. The availability of tangible, informational, and emotional support might be beneficial for meeting the support needs of carers [42, 43]. A local study has also shown that the sources of social support are important in influencing the perceived burden and needs of carers of palliative care patients, with stronger family support decreasing caregiver burden and needs [33]. Future research is recommended to examine which types and sources of social support are associated with the support needs of carers.

The study findings are in line with those from prior studies that showed excellent content validity and acceptability of the CSNAT [13, 14, 17], though the latter findings were previously and qualitatively derived. The S-CVI and the I-CVIs of the traditional Chinese version of the CSNAT far exceeded the recommended value of 0.78 [36], suggesting that the CSNAT items were very relevant and appropriate for the assessment of support needs among carers. The study also found that the CSNAT was highly acceptable to the HCPs and carers. In particular, the carers perceived the CSNAT as comprehensible and they felt comfortable completing it, and these findings are useful due to the nature of the self-assessment tool. Despite research that has identified carers and HCPs’ attitudes as one of the challenges in implementing the CSNAT during clinical practice [44, 45], the current study showed that both HCP and carers had a very high willingness to complete and use the CSNAT for needs assessment and support, providing an important basis for considering its future application in the local context.

### Implications

This study provides further psychometric data in support of the evidence-based CSNAT tool within the Hong Kong palliative care context. Successful validation of the CSNAT tool is an important first step before proceeding with the provision of person-centered care to meet the comprehensive support needs of carers. The CSNAT has been successfully integrated into an intervention with a five-stage process of carer assessment and support during palliative and end of life care [15]. Increasing evidence has shown the benefits of the CSNAT intervention in improving caregiver outcomes, including caregiver burden and quality of life, in the contexts of the United Kingdom and Australia [46, 47]. Future research should be considered to quantitatively and qualitatively explore the feasibility and effectiveness of the CSNAT intervention with local carers and HCPs.

### Limitations

Several limitations should be acknowledged before making its conclusion. First, as the participants were recruited from palliative care hospitals, the findings cannot be generalized to community-based care settings. Second, different mode of administration of the CSNAT at two assessment might introduce the respondent bias due to changes in the social setting (clinic vs. home), possibly influencing the interpretation of the test-retest reliability results [48]. Future researchers are suggested to use experimental or randomization methods to allocate the different questionnaire modes to participants and standardize the mode of administration; including timing and outcome explanation [48, 49]. Third, the study was restrained in its use of convenience sampling, resulting in a risk of selection bias and a limited generazability of study results.

## Conclusion

The traditional Chinese version of the CSNAT is a holistic screening tool with high validity and acceptability as well as acceptable reliability that measures family carers’ support needs in palliative care settings. Since the CSNAT is brief and easy for practical use, we recommend that this tool should be considered for wide application in local palliative care practices. Future intervention should be developed in the next step in terms of integrating the CSNAT as part of needs-based carer support intervention for family carers.

## Data Availability

The datasets used and/or analyzed during the current study are available from the corresponding author on reasonable request.

## Abbreviations

CSNAT: Carer Support Needs Assessment Tool
HCP: Healthcare provider
C-M-CSI: Traditional Chinese version of the Caregiver Strain Index
C-CGI-18: 18-item traditional Chinese version of the Caregiver Inventory
SSQ-6: 6-item Social Support Questionnaire
Kw: Weighted quadratic kappa
CVI: Content validity index
